# Prevalence of antibody to Hepatitis B core antigen and Hepatitis B virus DNA in HBsAg negative healthy blood donors

**DOI:** 10.1186/s12985-016-0492-8

**Published:** 2016-03-05

**Authors:** Gharib Karimi, Maryam Zadsar, Nasrin Vafaei, Zohreh Sharifi, Mohammad FalahTafti

**Affiliations:** Blood Transfusion Research Center, High Institute for Research and Education in Transfusion Medicine, Hemmat Exp. Way, Next to the Milad Tower, Tehran, Iran

**Keywords:** Hepatitis B virus, Blood Transfusion, Blood Donors, Occult hepatitis B virus, Blood safety

## Abstract

**Background:**

Hepatitis B virus is one of the most important blood born viruses. Although the sensitivity of screening tests has been considerably increased, transmission may still occur due to window period or occult hepatitis B infections (OBIs). This study was aimed at evaluating the prevalence of the anti-HBc and identifying the HBV DNA in HBsAg negative blood donors.

**Methods:**

The Blood samples from 2031 HBsAg-negative blood donors were divided into three aliquots and tested for anti-HBc, anti-HBs and HBV DNA. Serologic screening including anti-HBc and anti-HBs was performed. As a confirmatory test, all positive results for anti-HBc were retested with another kit. Two positive results were considered for anti-HBc positivity. All HBsAg negative selected donations were tested by PCR assay on pooled specimens (five samples per pool), plasma samples found to be HBsAg negative but anti-HBc positive were selected for a single-unit specimen Real-Time assay.

**Results:**

The study population had a mean age of 33.25 ± 10.09 years were mainly composed of males (94.8 %). The seroprevalance rate was 4.9 % for Anti-HBc and 31.9 % for HBsAb. The majority (58.6 %) of Anti-HBc positive cases were regular blood donors with 42–49 years being the largest age group (41.4 %). Neither individual NAT nor pooled NAT test detected any HBV DNA.

**Conclusion:**

However, Screening of anti-HBc Ab is proposed as a method to identify previous contact with HBV, but there is controversy in literature data regarding the cost-benefit of exclusion of positive anti-HBc Ab in blood donors. Our data does not suggest HBc-Ab test as a screening tool in the study setting.

## Background

Blood and blood products are inseparable part of the treatment in many medical settings. Therefore, the availability of adequate safe blood and blood products remains a major concern in health care system and transfusion practice. The limited data from WHO Global Database on blood safety indicates around 92 million blood donations worldwide and more than 9 million blood transfusions in ninety countries, annually [1]. Currently, Iran has achieved 100 % voluntary non-remunerated blood donation, and in 2011 about 2 million blood donations were recorded [2]. For a variety of the most important infectious agents that are transmitted via blood transfusion such as HBV, HCV, HIV and syphilis, screening tests are performed as routine practice. However, despite considerable improvements in eligibility criteria for blood donation and development of more advanced screening methods, transfusion transmitted infectious agents like hepatitis B virus still present as a threat for blood safety. Currently About 0.2 % of donated blood from apparently healthy donors in Iran is diagnosed as HBsAg-positive and is hence discarded [2]. This phenomenon is observed on different scales in other blood services across the world [3]. By perceiving the HBV host interaction, it was shown that even HBsAg negative individuals might be infected and there is a chance of transmission especially upon transfusion. Thus, despite all efforts including the use of a highly sensitive HBsAg test, transmission may still occur from apparently healthy blood donors. This may be attributed to the inability of the screening tests to detect HBsAg during a window period or as a result of the occult HBV infections (OBIs). OBI arises when the HBV DNA is detected, while HBsAg remaining undetectable. In about 20 % of cases the only positive marker is HBV DNA but in other situations anti-HBc or anti-HBs could be detected as well. Several factors may be involved in OBI, including mutated HBsAg, low level expression of HBsAg or entrapment of antigen in the circulatory immune complexes [4–6]. The prevalence of hepatitis B virus infection in the population and the sensitivity of laboratory methods could affect the reported prevalence rates of OBI [7]. Some studies have suggested that in HBsAg negative and anti-HBc positive cases, there is a possibility of low level infectious HBV viremia. This study was conducted in order to determine the frequency of anti-HBc and HBV DNA in blood donors with undetectable HBsAg. Since HBsAg test is the only screening method in Iranian blood donation centres, the necessity for supplemantary screening tests such as anti-HBc or NAT test were studied as well.

## Methods

During the time frame of the present study (2013), 86,182 blood donations from voluntary blood donors in two main blood collection centres (Kermanshah and Ahwaz) were evaluated for HBsAg. HBsAg positive donors were excluded and HBsAg negative donors were considered for inclusion in the study. The donors were categorized as first-time, repeated and regular donors on the basis of blood donation history and according to the definition by Iranian Blood Transfusion Organization (IBTO). Based on the calculated sample size, a total of 2031 HBsAg negative donations were randomly selected. The samples were centrifuged and plasma was separated. Each sample was divided into three aliquots and stored at −70 °C for further processing. The samples screened for anti-HBc, anti-HBs and HBV DNA according to standard procedures carried out in the laboratories of IBTO. This study was approved by the IBTO Ethics Committee. Anti-HBc positive and negative subjects were further studied in terms of demographic variables such as age, gender, occupational status, in addition to blood donation and vaccination history.

### ELISA tests

Serologic screening including anti-HBc (Cut-off = (NC + PC)/5; OD = 450 nm; positivity of > 1.1) and anti-HBs were tested (DIA.PRO Diagnostic Bioprobes s.r.l, Milano, Italy) according to the manufacturer’s instructions. All positive results for anti-HBc were retested with another kit (IgM-IgG-HBc Ab DADE Behring, Germany). Two positive results were considered for anti-HBc positivity.

### Detection of HBV DNA

All HBsAg negative selected donations were tested by PCR assay on pooled specimens (five samples per pool) plasma samples found to be HBsAg negative but anti-HBc positive were selected for a single-unit specimen Real-Time assay. HBV-DNA was extracted from 200 μl of plasma by using the QIAamp DSP virus purification kit (Qiagen GmbH, Hilden, Germany) and detected with a commercial PCR kit (Qiagen GmbH, 4 Hilden, Germany) on a Light Cycler instrument (Roche Diagnostics-version2) according to the protocols recommended by the manufacturers. In each run, we used plasma negative and positive samples to prevent false positive and negative results. Also, to verify both steps DNA extraction and inhibition in PCR reaction, internal control was used for all samples. The lower detection limit for Real-Time PCR in this study was 9 IU/mL. Also, for confirmation of the results obtained, another sensitive in house HBV-DNA NAT was used that the sensitivity of the assay for HBV-DNA was 30 IU/ml. The analytical sensitivity of the PCR test was assessed using triplicate analysis of 10-fold serial dilutions of the world health organization (WHO) international standard for HBV (NIBSC; Hertfordshire, United Kingdom). The 95 % detection limits of the assays were calculated by probit analysis. The lower limit of detection of the PCR was 20 IU/ml. Clinical specificity was determined using 100 blood donor samples that were negative for HBV DNA, resulting in a clinical specificity of 100 %. The clinical sensitivity of the PCR assay was determined using a low-titer dilution series of an HBV genotype D specimen. HBV DNA was diluted to concentrations of 150, 125, 100, 75, 40, 30, 20 and 10 IU/ml. The lower limit of detection of the PCR was 30 IU/ml. For detecting all of the HBV genotypes, WHO International Reference Panel, containing hepatitis B virus genotypes A–G was used for assays.

## Results

**Fig. 1:**
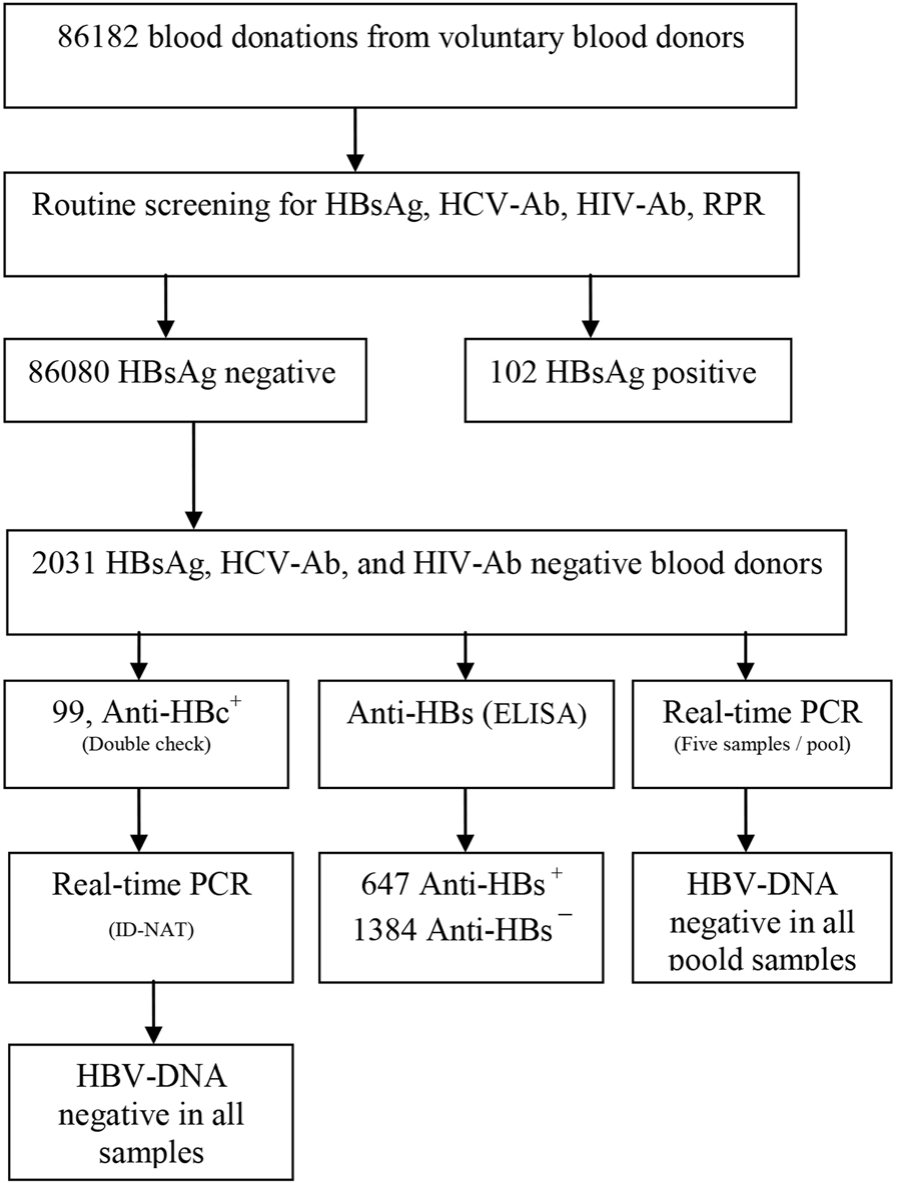
Flow chart of laboratory process in HBsAg-negative blood donations

During the study period, 86,182 units of blood from volunteer donors were collected. A total of 102 (0.12 %) blood donors were HBsAg positive and therefore were not further evaluated. As shown in flow-chart, the results of this study were obtained from three laboratory tests: Anti-HBc, Anti-HBs and NAT (Fig. 1). Anti-HBc positivity rate was 4.9 % (99/2031). The study population consisted of 1928 males (94.92 %) and 103 females (5.08 %), between 18 and 65 years with a mean age of 33.25 ± 10.09. The anti-HBs positivity rate was 31.9 % (647/2031) among the entire study population. It is reported a vaccination history in 591 (29 %) of blood donors of which 380 (64.3 %) were Anti-HBs positive. The majority (98.9 %) of Anti-HBc positive donors were male and mainly from the 42–49 year age group (41.4 %). There were statistically significant differences between Anti-HBc positive and negative donors in age groups (*p* = 0.001). In anti-HBc positive subjects, 15.2 % (15/99) were anti-HBs negative. Fifty-eight (58.6 %) of anti-HBc positive individuals were regular blood donor. statistically significant relationship was observed between anti-HBc positivity and regular blood donation (*P* = 0.001). All of the 99 anti-HBc positive samples that were tested by individual nucleic acid test were HBV DNA negative. Moreover, pooled NAT (five samples per pool) on the HBsAg negative samples (2031) did not show HBV DNA in any sample (Table 1).

**Table 1:** Serological prevalence and other characteristics among blood donors

		Serological profile				Total
Characteristics		HBsAb +	HBsAb +	HBsAb –	HBsAb –	
		HBcAb +	HBcAb –	HBcAb +	HBcAb –	
		84 (4.1 %)	563 (27.76 %)	15 (0.74 %)	1369 (67.4 %)	2031
Mean age ± SD		42.6 ± 9.95	35.3 ± 10.4	39.6 ± 11.8	34.6 ± 8.9	-
Donor type	First time	16 (19 %)	82 (14.6 %)	7 (46.7 %)	300 (22 %)	405
	Repeated	16 (19 %)	128 (22.7 %)	2 (13.3 %)	290 (21.1 %)	436
	Regular	52 (62 %)	353 (62.7 %)	6 (40 %)	779 (56.9 %)	1190
Vaccination history		30/84 (35.7 %)	350/563 (62.2 %)	4/15 (26.7 %)	207/1365 (15.2 %)	591

## Discussion

Detection of hepatitis B virus in blood donors with Australia antigen test (HBsAg test) began in 1970. In respect to the above mentioned issue, Iranian Blood Transfusion Organization since its establishment in 1974 has screened all donated blood for hepatitis B virus.

In 1979, the prevalence of HBsAg among voluntary blood donors in Iran was reported to be 3.4 % [8]. However this rate has been dramatically decreased over the past 30–35 years and the current prevalence rate of HBsAg among voluntary blood donors has reached to 0.2 % [2], with variations between different regions. Furthermore, HBV prevalence rate of 2.14 % has been reported in the general population [9]. This decrease could be attributed to the improvement in donor selection procedures along with the application of community based preventive measures such as integration of HBV vaccination in Expanded Programme on Immunization (EPI) since 1993, increased community health education, and development of highly sensitive screening tests. Owing to these safety strategies post transfusion hepatitis is now a very rare event. No evidence about the incidence of post transfusion hepatitis B in Iran has been found. In a review article published by H. Rezvan et al. the prevalence of HBSAg in β thalassemia major and haemodialysis patients (as multitransfused patients) has been reported around the general population [10]. Even though, there were no evidence that they are actually infected via blood transfusions. So that, we are not capable to find any evidence in case of post transfusion hepatits B prevalence in Iran. Nevertheless, circulating HBV DNA in HBsAg negative blood donors (occult HBV infection) can be a threat to blood safety. Therefore, performing HBsAg test does not eliminate the risk of HBV transmission to blood recipients completely. It has been reported that OBI is associated with the presence of anti-HBc or anti-HBs. Even more, in some cases neither anti-HBc can be detected nor anti-HBs [11]. Given the above considerations, it is proposed that implementing genomic screening (NAT) in high and low prevalence countries could lead to substantial safety in view of window period and OBI related risk of transfusion transmitted Hepatitis B. The detection limit of NAT ranges from 1:1000 to 1:50,000 depending on the epidemiology and sensitivity of the assay as described by Alain [3].

The rate of positive serologic test results for anti-HBc was found to be 4.9 % in the present study and isolated anti-HBc were found in 15 cases (0.74 %). In the literature, anti-HBc prevalence rates between 2.1 and 11.5 % has been reported among Iranian blood donors (Table 2), and the prevalence of anti-HBc in Iranian general population has been reported to be 16.4 % [22]. Thus, our results are in accordance with the literature data. The slight differences between our results and the previous ones may be described by many factors such as different sample size, laboratory methods, and different epidemiological conditions.

**Table 2:** Reported prevalence of Anti-HBc, HBV DNA and OBI in HBsAg Negative blood donors based on some published studies in Iran

Reference	City	Number of donors (HBsAg^-^)	Anti-HBc^+^ n (%)	HBV DNA^+^/Anti-HBc^+^ n (%)	OBI n (%)
Sofian et al. [12]	Arak	531	11(2.1 %)	0/11 (0 %)	0
Shahabi et al. ^a^	Mashhad	5059	432(8.5 %)	0/60 (0 %)	0
AminiKafi-abad et al. [13]	Tehran	2000	230 (11.5)	0/230 (0 %)	0
Khamesipour et al. [14]	Rasht	2041	78 (3.8 %)	1/78 (1.3 %)	1 (0.05 %)
Pourazar et al. [15]	Isfahan	545	43 (8 %)	5/43 (11.62 %)	5 (0.92 %)
Behbahani et al. [16]	Shiraz	2000	131 (6.55 %)	16/131 (12.2 %)	16 (0.8 %)
Arababadi et al. [17] ^a^	Rafsanjan	3700	352 (9.51 %)	57/352 (16.1 %)	57 (1.54 %)
Jafarzadeh et al. [18]	Rafsanjan	270	14 (5.18 %)	4/14 (28.57 %)	4 (1.48 %)
Delavari et al. [19]	Kerman	1525	121 (8 %)	36/121 (29.7 %)	36 (2.36 %)
Vaezjalali et al. [20]	Tehran	1000	80 (8 %)	40/80 (50 %)	40 (4 %)
Alizadeh et al. [21]	Tehran	5000	499 (9.8 %)	2/499 (0.4 %)	2 (0.04 %)
Total	–	23,671	1991 (8.4 %)	161 (9.9 %)	161 (0.7 %)

^a^ Article published in Persian

Several conditions may result in positive isolated anti-HBc in an apparently healthy blood donor. One possibility is recovered past HBV infection, which did not apply to our donors as they had no history of hepatitis. Nevertheless, hepatitis B infections are sometimes asymptomatic and patients do not manifest jaundice or any other symptoms/signs. Another possibility is false-positive results due to be poor specificity of the test [23]. To minimize false-positive results, we repeated the test with the same method, and all reactive samples were positive in repeated measurements. Finally, there is risk of HBV transmission in pre-seroconversion widow period, i.e. a period that infected blood donors have not been seroconvert yet, hence HBsAg and anti-HBc are negative. Therefore, in addition to anti-HBc positive cases, all HBsAg negative samples were also evaluated by minipool nucleic acid testing. Our study demonstrated an anti-HBs prevalence rate of 31.9 % (647/2031) in HBsAg negative blood donors. Specimens from 84 blood donors were positive for both anti-HBs and anti-HBc. Thus, 563 blood donors were positive for anti-HBs alone. Regarding the vaccination history of 591 cases, it appears that in spite of vaccination, 28 subjects could not produce the HBsAb response.

Considering the fact that concurrent detection of anti-HBc and anti-HBs might be assessed as an indicator of past exposure and recovery, it can be inferred from our results that 4.1 % of our donors had a history of infection, and were immune due to previous exposure/infection. There is evidence that HBV DNA can be detected even in anti-HBs positive blood donors [24, 25]. Thus it has been suggested that the presence of anti-HBs antibody does not rule out the possibility of HBV transmission. In this study, no HBV DNA in the serum samples of HBsAg negative blood donors was detected regardless of positive or negative anti-HBc and anti-HBs test results. A review of the similar published studies in Iranian blood donors shows different statistics. According to a review of 11 published articles, the prevalence of HBV DNA in HBsAg negative/anti-HBc positive blood donors varied from 0 to 50 % (Table 2). These studies have been carried out in different geographical areas and also have used different PCR methods such as qualitative, real time and nested PCR. Our study as well as three other studies suggests the absence of OBI in the studied blood donors. Therefore it may be suggested that these results may be influenced by methods and/or laboratory conditions (sampling condition, testing methods and equipment) used by different researchers. Vaezjalali et al, who reported the highest frequency, claimed improved accuracy of HBV DNA detection through the use of a homemade nested PCR technique with at least two different sets of primers in their study. In a study conducted earlier by Shahabi et al (article in Persian) this method has been used as well. In the mentioned article, there was no evidence of HBV DNA in 60 samples.

The results of similar studies on the frequency of HBV-DNA positivity in HBsAg negative, anti-HBc positive blood donors in other countries show great variation. The frequency was reported 0 % in Turkey and Greece [26–28], 1.25 % in Saudi Arabia [29], 1.59 % in Germany [23], 4.86 % in Italy [30], 7.5 % in India [31], 17.2 % in Egypt [32], 38 % in Japan [33] and 90.5 % in Sudan [34].

Differences in the frequencies of occult HBV infection in different studies could be related to the endemicity of HBV infection, geographical variations, the sensitivity of the screening tests being used to detect HBV DNA and other viral markers, sample size, power of the study, composition of study population and so on.

Evidence shows that anti-HBc testing has only a little effect on the blood safety. However, the use of individual NAT test can improve the HBV related blood safety. In spite of introducing the individual NAT test, transfusion centres cannot bypass the serological tests and various combinations of serology and NAT are implemented in different countries [3]. Currently, about 27 countries in the world have implemented NAT for hepatitis B virus screening in blood donors as a mandatory or voluntary approach [35]. High coverage of HBV vaccination in the early infantile period (around 97 %) was achieved and consequently a marked decrease in the HBV prevalence among general population was reported. In addition, vaccination in the health care workers has been implemented since 1993 and mass vaccination for adolescents who was born from 1989 to 1992 was also performed to extend the immunity to larger group of young population. Moreover, a series of studies on the general population have shown that the efficacy of hepatitis B vaccine has been more than 80 % [36, 37]. The combined effect of vaccine and improvement on public health status resulted change in the HBV epidemic and Iran now is in a low-intermediate state. So that the epidemiological feature of virus in Iran might be presented as a describing factor to decrease the necessity of NAT test on Iranian blood donors.

## Conclusions

Occult HBV infection is a complex issue especially given the fact that many circumstances and conditions may influence the clinical and laboratory findings. In order to detect HBV-DNA in very low levels, it is necessary to use a highly sensitive technique.

In this study, HBV-DNA in anti-HBc seropositive blood donors could not be detected. Therefore, based on our data it is not suggested to screen anti-HBc or HBV NAT in Iranian blood donors. On the other hand, different rates of occult HBV infection in blood donors are reported across the country. In addition it should be considered that anti-HBc screening of the blood donors will lead to the loss of a significant number of donors. For example, in our experience, screening the donors for anti-HBc could result in approximately 5 % loss in blood donations, without significant evidence that such condition could affect the blood safety. Therefore, the decision on implementing the anti-HBc and HBV NAT should be based on more comprehensive, multicenter studies to perceive the actual amount of occult HBV infection in the general population and blood donors.
